# Delayed diagnostic imaging but stable treatment initiation for kidney cancer during the COVID-19 pandemic: a Hungarian cohort study

**DOI:** 10.3389/pore.2026.1612411

**Published:** 2026-05-13

**Authors:** László Rumi, Árpád Szántó, Dániel Bányai, Éva Szabó, Szabolcs Bellyei, Dóra Hubai, Balázs Takáts, Tamás Bodnár, János Girán, István Kiss, Árpád Boronkai, Éva Pozsgai

**Affiliations:** 1 Urology Clinic, University of Pécs Clinical Center, Pécs, Hungary; 2 Department of Otorhinolaryngology, University of Pécs Clinical Center, Pécs, Hungary; 3 Department of Oncotherapy, University of Pécs Clinical Center, Pécs, Hungary; 4 Klinik für Anästhesie, Luzerner Kantonsspital, Sursee, Switzerland; 5 Department of Public Health Medicine, University of Pécs Medical School, Pécs, Hungary; 6 Clinical and Health Data Analytics Lab, Department of Public Health Medicine, University of Pécs Medical School, Pécs, Hungary; 7 Department of Primary Health Care, University of Pécs Medical School, Pécs, Hungary; 8 Department of Otorhinolaryngology Head and Neck Surgery, Szent György University Teaching Hospital, Székesfehérvár, Hungary

**Keywords:** advanced-stage, COVID-19, kidney cancer, predictive factor, renal cell carcinoma, waiting time, time to treatment, time to diagnosis

## Abstract

**Background/Objectives:**

The COVID-19 pandemic influenced cancer care worldwide, delaying diagnosis and treatment. We compared waiting times — time to initial diagnostic imaging, time to treatment, time to histopathological diagnosis, and length of hospital stay — of kidney cancer patients between pre-COVID-19 and COVID-19 periods at a large regional Hungarian clinical center. We also aimed to identify factors predicting prolonged waiting times.

**Methods:**

Data from 400 adult kidney cancer patients (all histologically renal cell carcinoma) at the University of Pécs Urology Clinic were analyzed retrospectively, for two periods (1 January 2019–15 March 2020, pre-pandemic; 16 March 2020–13 May 2021, pandemic). Demographic and clinical characteristics were collected, and time intervals calculated from electronic health records, followed by statistical analyses.

**Results:**

Median time from symptom onset to initial diagnostic imaging increased significantly from 7.5 to 34 days during the pandemic (p = 0.026), while time to treatment (p = 0.492), time to histopathological diagnosis (p = 0.575), and length of hospital stay (p = 0.319) remained stable. Median healthcare-related waiting times (time to treatment and time to histopathological diagnosis) were comparatively long (range: 95.5–111 days). Advanced-stage disease (III–IV) was protective for prolonged time to initial diagnostic imaging (OR 0.205, 95% CI 0.074–0.568) pre-pandemic and for prolonged time to histopathological diagnosis (OR 0.496, 95% CI 0.254–0.971) during the pandemic. No other demographic or clinical factors influenced waiting times significantly.

**Conclusion:**

The pandemic prolonged the interval from symptom onset to initial diagnostic testing, likely due to fear-driven healthcare avoidance by patients, but did not affect healthcare-related waiting times. Advanced-stage disease predicted shorter waiting times, with variable influence across periods. Our findings highlight the need for patient education and careful prioritization of care, with waiting times exceeding those reported in other international settings.

## Introduction

The COVID-19 pandemic affected cancer care worldwide, including kidney cancer (KC) management, with studies reporting delays in both diagnosis and treatment [1–4]. These delays have been linked to changes in stage at diagnosis, prolonged waiting times, and poorer cancer-specific survival [1–3, 5, 6]. In Hungary, lockdown measures and the prioritization of COVID-19 patients similarly disrupted cancer services [7, 8].

Kidney cancer ranks as the third most common urological malignancy worldwide and renal cell carcinoma (RCC), which accounts for approximately 90% of kidney tumors, represents the main histological subtype [9]. KC shows marked gender differences, ranking as the fourteenth most frequent cancer in women and the ninth in men [9–11].The disease is often detected incidentally, with up to 85% of localized renal masses discovered during imaging for unrelated conditions [12]. Less than a third of RCC cases present with clinical symptoms, as small tumors are often asymptomatic [13]. Mortality from KC has paradoxically increased in certain European countries despite improvements in the disease’s management in recent decades [14]. Timely diagnosis is crucial, as prognosis depends on stage at presentation, with overall survival declining from 90% for localized disease to around 12% for patients with distant metastases [11, 15–17].

Delays in establishing a kidney cancer diagnosis and ultimately treatment arise from both patient- and healthcare-related factors. Patients may be asymptomatic or may overlook symptoms, contributing to patient-related delays [18]. Additional barriers may stem from healthcare-related delays — for example, limited diagnostic capacity, workforce shortages, long waiting lists for imaging or specialist services, and under-resourced hospitals- which ultimately influence clinical outcome [17, 19].

As a result of the pandemic both patients’ healthcare-seeking behavior and the organization of cancer care were affected [5, 20, 21]. Many people postponed cancer screening tests and delayed routine medical appointments due to fear of infection and limited access to services [21, 22]. During the pandemic, studies reported increased surgical wait times and higher rates of advanced or metastatic RCC. Janes et al. reported an increase in surgical wait times from 44.5 days pre-pandemic to 56.8 days during the pandemic (p = 0.003) [6]. A study in India reported a mean surgical delay of approximately 15 days for RCC patients attributed to COVID-19-related procedural changes, which prolonged overall treatment duration and increased costs [1]. A significant increase in metastatic RCC cases (13.1% vs. 6.1%, p = 0.01) and longer waiting times between imaging and surgery (35 vs. 30 days, p = 0.01) was reported in Turkey during this period [23].

There is limited data from the Central and Eastern European region on the effect of the pandemic on kidney cancer waiting times and the possible influencing factors of waiting times compared to Western countries [8]. In a recent study we reported changes in kidney cancer detection patterns and clinical characteristics during the pandemic [8], however to our knowledge, no studies have examined waiting times in this region.

Therefore, the aim of our study was to assess and compare the waiting times to initial diagnostic testing, initiation of treatment, and histopathological diagnosis as well as length of hospital stay among individuals diagnosed with KC during the pre-pandemic and pandemic periods. Furthermore, we aimed to identify potential predictors of prolonged waiting times across the two study intervals.

## Methods

### Setting

Ethical approval for this study was provided by the Regional Ethical Committee of the University of Pécs (Reference No. 9389 – PTE 2022) prior to the initiation of data collection and analysis.

The study was carried out at the Urology Clinic of the University of Pécs Clinical Center (UC), located in Pécs, Hungary. The UC serves as a principal referral center for urologic oncology across Baranya County and adjacent Transdanubian regions. Conducting approximately 4,000 surgeries each year, the clinic has established recognized expertise in nephron-sparing approaches for renal cancer.

### Study design and patient inclusion criteria

This investigation followed an observational, retrospective design. Eligible participants included adult patients (≥18 years) who were treated at the UC between 1 January 2019, and 13 May 2021, and had a histologically verified diagnosis of renal cell carcinoma, recorded with the ICD-10 code C64. Exclusion criteria included individuals with any malignant neoplasm diagnosed within the preceding 5 years of the study or those with secondary tumors.

The study timeframe was stratified into two distinct phases corresponding to the national pandemic timeline, defined according to directives from the Hungarian National Directorate General for Hospitals, reflecting changes in healthcare service provision during the pandemic.

The two study periods were: Pre-COVID-19 period: 1 January 2019 – 15 March 2020; and COVID-19 period: 16 March 2020 – 13 May 2021.

### Data collection and variables

Data extraction was performed from the electronic health records of the University of Pécs Clinical Center. Automated database screening identified all patients fulfilling the inclusion criteria, yielding a cohort of 400 individuals diagnosed with kidney cancer during the two study periods.

Demographic data collected included age at presentation, sex, and residential location. Manual chart review supplemented this with variables such as distance from the patient’s residence to the UC (calculated via Google Maps using the shortest driving route), comorbidities, initial symptoms, tumor stage, and mode of cancer detection (symptomatic vs. asymptomatic).

Comorbidities were recorded following the Charlson Comorbidity Index (CCI) framework and categorized into two risk groups: low/moderate (0–4 points) and high (≥5 points), reflecting predicted 10-year survival. Asymptomatic cases were defined as patients whose renal cancer was incidentally discovered during imaging (ultrasound, CT, MRI, or X-ray) conducted for unrelated medical conditions. Symptomatic cases were those presenting with clinical manifestations (including haematuria, flank/abdominal pain, weight loss/sweating, cough/mass) attributable to kidney cancer. Diagnostic codes and comorbidities were classified using the ICD-10 system to ensure standardized reporting.

### Definition of dedicated dates and investigated time intervals

The date of symptom onset was determined according to the patient’s subjective opinion of when their symptoms started as stated in their medical records.

The date of initial diagnostic imaging was the date the patient first underwent diagnostic imaging raising clinical suspicion of KC.

The date of initial treatment was defined as the date the patient started therapy. In almost all cases this meant the day of surgery; in six pre-pandemic and eight pandemic cases, this referred to other oncologic treatments (Table 1C).

**TABLE 1:** Baseline characteristics of KC patients (N = 400) attending the UC before and during the COVID-19 period [8]. A) Demographic characteristics B) Clinical characteristics C) Symptom presence and management.

A)	Before COVID-19		During COVID-19		Chi-square Test’s p value
	n	*%*	n	*%*	
Sex	​	​	​	​	0.023
Male Female Total	139	*68.1*	112	*57.1*	​
	65	*31.9*	84	*42.9*	​
	204	*100.0*	196	*100.0*	​
Age	​	​	​	​	0.307
0–49 50–59 60–69 ≥ 70 Total	30	*14.7*	27	*13.8*	​
	54	*26.5*	57	*29.1*	​
	62	*30.4*	71	*36.2*	​
	58	*28.4*	41	*20.9*	​
	204	*100.0*	196	*100.0*	​
Place of residence	​	​	​	​	0.087
County seat City Other location Total	54	*26.5*	54	*27.6*	​
	60	*29.4*	75	*38.3*	​
	90	*44.1*	67	*34.1*	​
	204	*100.0*	196	*100.0*	​
Distance from UP UC [km]	​	​	​	​	0.048
≤ 40 >0.40 Total	84	*41.2*	100	*51.0*	​
	120	*58.8*	96	*49.0*	​
	204	*100.0*	196	*100.0*	​

B)	Before COVID-19		During COVID-19		Chi-square Test’s p value
	n	*%*	n	*%*	
Charlson comorbidity index	​	​	​	​	0.048
≤ 4 ≥ 5 Total	86	*42.2*	102	*52.0*	​
	118	*57.8*	94	*48.0*	​
	204	*100.0*	196	*100.0*	​
Stage	​	​	​	​	0.632
I II III IV Total	151	*74.0*	135	*68.9*	​
	9	*4.4*	12	*6.1*	​
	32	*15.7*	38	*19.4*	​
	12	*5.9*	11	*5.6*	​
	204	*100.0*	196	*100.0*	​
Stage	​	​	​	​	0.417
I – II II – III Total	160	*78.4*	147	*75.0*	​
	44	*21.6*	49	*25.0*	​
	204	*100.0*	196	*100.0*	​

C)	Before COVID-19		During COVID-19		Chi-square Test’s p value
	n	*%*	n	*%*	
Type of initial imaging test	​	​	​	​	0.088
US CT MRI CEUS Plain chest X-ray Total	157	*77.0*	126	*64.4*	​
	36	*17.6*	52	*26.5*	​
	7	*3.4*	13	*6.6*	​
	3	*1.5*	4	*2.0*	​
	1	*0.5*	1	*0.5*	​
	204	*100.0*	196	*100.0*	​
Presence of symptoms	​	​	​	​	0.166
Symptomatic Asymptomatic Total	29	*14.2*	38	*19*.*4*	​
	175	*85.8*	158	*80.6*	​
	204	*100.0*	196	*100.0*	​
Radicality of surgery	​	​	​	​	0.169
Partial nephrectomy Radical nephrectomy Total***	128	*65.6*	110	*58.8*	​
	67	*34.4*	77	*41.2*	​
	195	*100.0*	187	*100.0*	​

* US: ultrasound.

** CEUS: Contrast-Enhanced Ultrasound.

*** Total is lower than the overall number of patients, as not all patients underwent surgery (Pre-pandemic: 6 received non-surgical oncologic care, 3 died before therapy; Pandemic: 8 received non-surgical oncologic care, 1 died before therapy).

The date of histopathological diagnosis was the date the cancer of the kidney (renal cell carcinoma) was histologically confirmed.

The investigated intervals were defined as follows:

Time to Diagnostic Imaging (TDI): number of days from symptom onset to initial diagnostic imaging (symptomatic patients only).

Time to Treatment Interval (TTI): number of days from initial diagnostic imaging to initiation of therapy (all patients).

Time to Histopathological Diagnosis (THI): number of days from initial diagnostic imaging to histopathological confirmation (all patients).

Length of Hospitalization (LOH): number of days from the start of therapy (most often surgery) to discharge.

It should be noted that in KC, since initial therapy almost invariably preceded histopathological confirmation, waiting times to histopathological diagnosis were generally longer than those to initial therapy.

**FIGURE 1:**
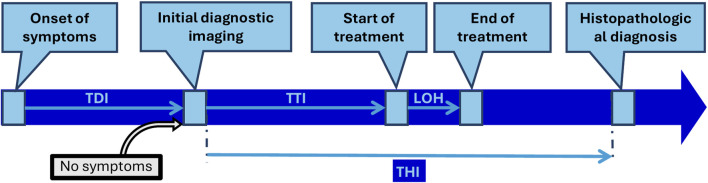
Investigated waiting times (TDI, TTI, THI) and LOH of KC patients in the study.

Figure 1 shows the investigated time intervals (Figure 1).

The primary outcomes of this study were to determine and compare waiting times among individuals diagnosed with KC in the pre-pandemic and pandemic periods. The secondary outcomes involved identifying potential predictors of prolonged waiting times across the two study intervals.

The potential predictive factors of waiting times examined are presented in Table 1A,B [8].

The baseline characteristics of the patient cohort have been described previously [8] and are summarized in Table 1A–C. Briefly, during the pandemic period, a higher proportion of female patients was observed, whereas the proportions of patients with a high Charlson Comorbidity Index (CCI) and those residing farther from the Clinical Center were lower. No significant differences between periods were reported for age distribution, residence type, initial imaging modality, cancer stage (early vs. advanced), or surgical approach [8] (Tables 1A–C).

### Data analysis

To address the study objectives, descriptive and inferential statistical analyses were performed. Frequency tables were utilized to characterize the demographic, clinical and treatment profiles of the patients. To examine the stochastic nature of the relationships between the analyzed data, we used the chi-square test, with p ≤ 0.05. For the analysis of the median values of the waiting times we employed the Mann-Whitney test. Logistic regression analysis was carried out to analyze the impact of demographic and clinical factors on the waiting times. For statistical analysis, all waiting-time intervals were categorized into two groups—greater than the median and median or less. Statistical analyses were conducted using Jamovi 2.6.26.

## Results

### Comparison of waiting times (TDI, TTI, THI) and LOH of KC patients between the two study periods

We analyzed the waiting times (TDI, TTI, THI) and LOH of KC patients between the pre-pandemic and pandemic periods. Table 2 presents the median values of waiting times (TDI, THI, TTI). and LOH (Table 2).

**TABLE 2:** Median waiting times of patients with KC before and during the COVID-19 period.

​	Before COVID-19 Median (Q1–Q3)	During COVID-19 Median (Q1–Q3)	p value Mann-whitney U test
Median time to diagnostic imaging _TDI_ [days]	7.5 (1.0–100.0)	34.0 (13.0–152.5)	0.026*
Median time from imaging to treatment _TTI_ [days]	101.0 (74.0–142.0)	95.5 (61.0–158.0)	0.492
Median time from imaging to histopathological diagnosis _THI_ [days]	111.0 (79.0–153.0)	105.0 (69.0–166.5)	0.575
Median time of hospitalization _LOH_ [days]	4.0 (3.0–6.0)	4.0 (4.0–6.0)	0.319

The median time from the onset of symptoms to initial diagnostic imaging (TDI) increased significantly, from 7.5 to 34 days during the pandemic (p = 0.026).

The median time from initial diagnostic imaging to treatment (TTI) and from initial diagnostic imaging to histopathological diagnosis (THI) among KC patients did not change significantly during the pandemic, although both were slightly shorter during the pandemic (TTI: 101 vs. 95.5 days, p = 0.492; THI: 111 vs. 105 days, p = 0.575).

The median length of hospital stays (LOH) showed no significant difference either, remaining at 4 days in both study periods (p = 0.319).

**FIGURE 2:**
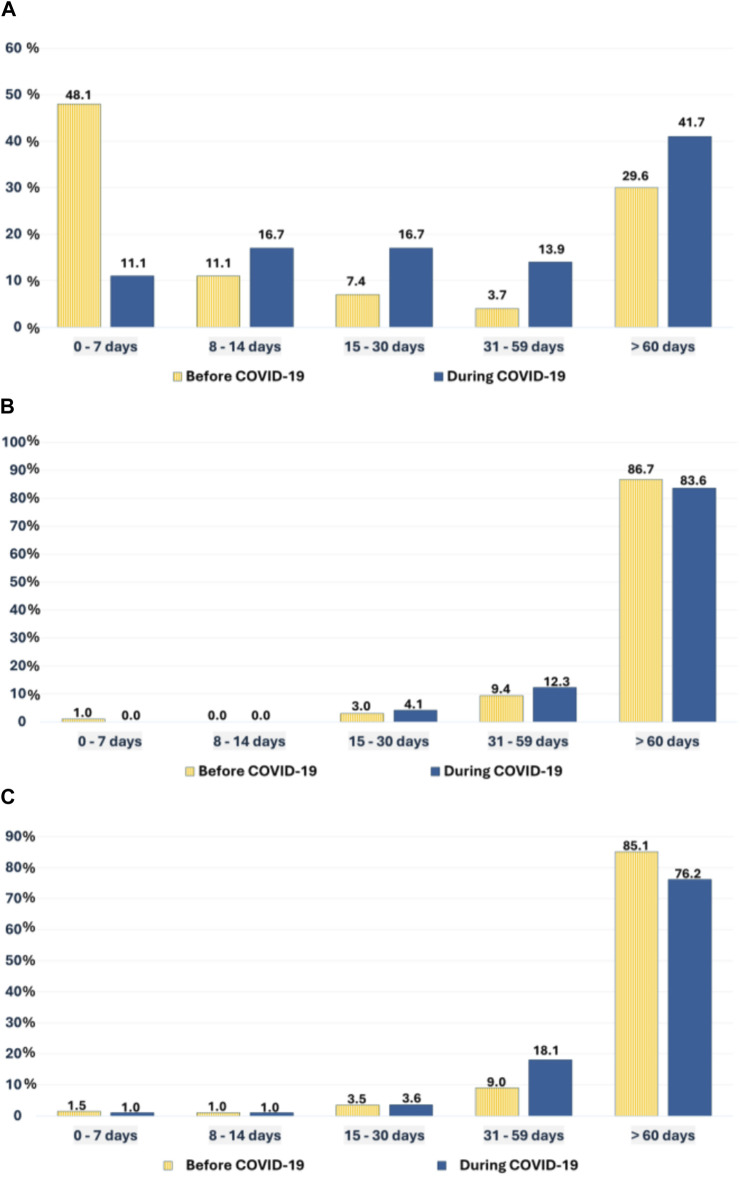
The distribution of waiting times TDI **(A)**, TTI **(B)** and THI **(C)** of KC patients in the two study periods.

Figure 2 illustrates the distribution of waiting times. A higher proportion of patients during the pandemic experienced TDI >60 days, whereas TTI and THI distributions were similar in the two periods (Figures 2A–C).

### Comparison of the effect of patient characteristics on waiting times (TDI, TTI, THI) and LOH

When comparing demographic (sex, age, residence type, distance from clinical center) and clinical (CCI score and stage) factors across the two study periods, only cancer stage showed a statistically significant association with waiting times, while no relationship was observed with LOH.

**FIGURE 3:**
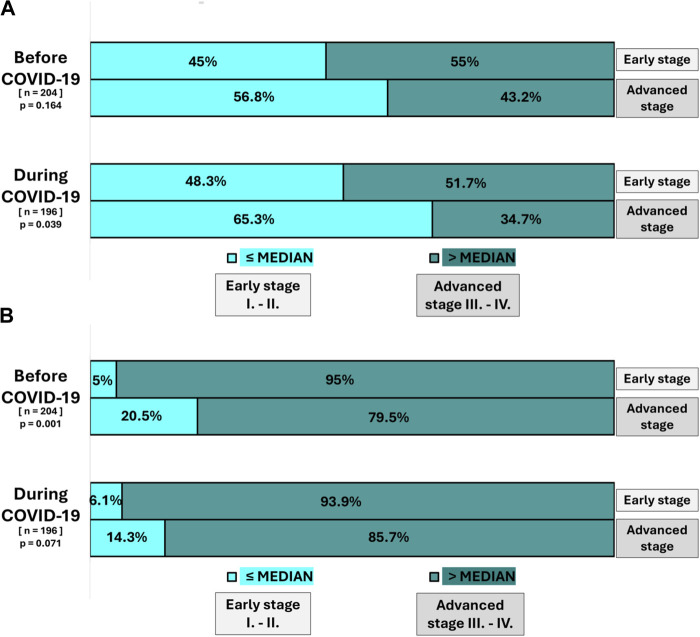
Factors showing statistically significant associations with TDI and THI waiting times during the pre-pandemic and pandemic periods **(A,B)**.

In the pre-pandemic period, a significantly larger proportion of advanced-stage patients (stage III–IV) had a TDI ≤ median (20.5%) compared with early-stage patients (5%) (p = 0.001). This association was not statistically significant during the COVID-19 period (6.1% early-stage vs. 14.3% advanced-stage; p = 0.071) (Figure 3A).

During the pandemic, a significantly larger proportion of advanced-stage patients (65.3%) had a THI ≤ median compared with early-stage patients (48.3%) (p = 0.039), however the association was not statistically significant for the pre-pandemic period (45% vs. 56.8%, p = 0.164) (Figure 3B).

No statistically significant differences were observed between early- and advanced-stage disease for TTI ≤ median in either period (pre-pandemic p = 0.143; pandemic p = 0.186) (Supplementary Table S1).

No statistically significant associations were observed between other demographic and clinical factors investigated (gender, age, distance from the Clinical Center, Charlson Comorbidity Index (CCI) score) and any of the examined time intervals (TDI, TTI, THI and LOH) as shown in Supplementary Table S1 (Figures 3A,B).

### Advanced cancer as a predictive factor for waiting times before and during the COVID-19 pandemic

Logistic regression analysis demonstrated that advanced tumor stage independently predicted the length of certain waiting times.

Pre-pandemic, patients with advanced-stage disease had significantly lower odds of experiencing longer-than-median TDI (OR = 0.205; 95% CI: 0.074–0.568), whereas this association was not present during the pandemic (Table 3).

**TABLE 3:** Assessment of advanced cancer stage as a predictive factor for longer waiting times (TDI, THI, TTI) and LOH in the study periods before and during the COVID-19 pandemic.

​	​	Before COVID-19 OR/CI 95%	During COVID-19 OR/CI 95%
TDI > median	Advanced-stage (III-IV) cancer	0.205 [0.074–0.568]	0.391 [0.137–1.114]
TTI > median	Advanced-stage (III-IV) cancer	0.606 [0.039–1.189]	0.642 [0.332–1.241]
THI > median	Advanced-stage (III-IV) cancer	0.622 [0.317–1.219]	0.496 [0.254–0.971]
LOH > median	Advanced-stage (III-IV) cancer	1.696 [0.863–3.330]	1.057 [0.551–2.025]

During the pandemic, advanced-stage disease was likewise associated with statistically significantly lower odds of experiencing longer-than-median THI (OR = 0.496; 95% CI: 0.254–0.971), a pattern not observed in the pre-pandemic period (Table 3).

Tumor stage was not a predictive factor for TTI or LOH in either period.

## Discussion

This study is among the few from the region to investigate kidney cancer waiting times and analyze the impact of the COVID-19 pandemic. We found that while median time to initial diagnostic testing (TDI) among symptomatic patients increased during the pandemic, time to treatment (TTI) and time to histopathological diagnosis (THI) remained stable. Advanced-stage disease was identified as a protective predictor against prolonged TDI in the pre-pandemic period and against prolonged THI during the pandemic.

Delays in care can be categorized as patient-related or healthcare-related. Patient-related delays—i.e., the interval until consulting a physician—often stem from personal decision-making barriers, or limited healthcare accessibility (e.g., appointment availability and diagnostic capacity) [24]. These factors make them a major contributor to overall diagnostic delay [24]. Survey data from an NCI-designated cancer center showed that during the COVID-19 pandemic delays in cancer-related care and preventive screening were common, particularly among patients with limited health literacy [21]. Furthermore, many patients postponed routine medical visits and cancer screening due to fear of infection and pandemic-related restrictions [20]. Our finding that TDI among symptomatic patients increased by 26 days during the pandemic suggests that similar phenomena may have taken place in the studied cohort.

The pandemic led to a sharp reduction in routine health checks and imaging, resulting in fewer incidentally detected small renal masses and a relative rise in symptomatic and advanced-stage presentations [2, 23]. Across Europe, North America, and Asia, this “stage migration” was consistently observed [1, 2]. More RCC patients presented with T3 or metastatic disease and symptomatic manifestations such as hematuria, rather than incidental findings [1, 2, 23]. The pandemic also substantially prolonged waiting times globally: a Canadian study found mean surgical delays increasing from 44.5 to 56.8 days (p = 0.003) during the pandemic [6], while Keskin et al. (2025) in Turkey reported fewer RCC surgeries, longer imaging-to-operation times (30–35 days; p = 0.01), larger tumors, and more metastatic cases in this period [25]. In India, a mean surgical delay of 15.19 days was reported and attributed to preoperative COVID-19 testing and related measures [1]. An investigation in Turkey also showed metastatic RCC rates nearly doubling (13.1% vs. 6.1%; p = 0.01) and longer imaging-to-operation times (35 vs. 30 days; p = 0.01) [23]. However, some reports have found no significant changes in time to treatment. According to a study in the Netherlands, median time from radiological diagnosis to surgical treatment was on average 48 days prior to the pandemic, and 46 days during the first wave of the pandemic [26]. Consistent with these findings, median TTI and THI did not change significantly in our cohort either. This suggests that the primary pandemic impact occurred before healthcare entry, as reflected by prolonged TDI. Once patients accessed the system, care pathways were largely maintained. It should be noted, however, that approximately 10% fewer KC cases were observed during the pandemic, primarily due to reduced incidental diagnoses, which may have decreased system burden and allowed routine cases to proceed without delay [8].

Median THI and TTI values (ranging between 95.5 and 111 days) in our study were longer than internationally reported values [3, 23, 26]. In Canada, a multicenter cohort study reported a median time from initial consultation to nephrectomy of 41 days, with stage-specific variation ranging from approximately 30–57 days depending on tumor stage [27]. Substantially shorter surgical pathways were demonstrated in a study in China, where median time from diagnosis to surgery was 16 days [28]. In cohorts from European and neighboring countries, median waiting times have been reported to be slightly longer, ranging between 30 and 48 days [23, 26].

Several factors may underlie the longer waiting times observed in our study. Although Hungarian law mandates CT or MRI within 14 days of cancer suspicion, scan interpretation can take an additional 4–10 days due to radiology staffing shortages [29]. Limited oncology specialists and surgical capacity outside major centers may also contribute to delays [29]. The Healthy Budapest Program for example reduced imaging waiting times, but intervals from diagnosis to therapy remained prolonged, likely due to referral processing, tumor board scheduling, preoperative evaluations, and operating room coordination [30]. These bottlenecks can cumulatively extend the time from diagnosis to surgery, reflecting systemic access challenges in Hungary [30]. Improving KC pathways would be particularly important, since waiting times exceeding 3 months have been associated with worse outcomes in RCC [17, 19]. In patients with T1b–T2a disease, delayed surgery exceeding approximately 90 days has been associated with decreased cancer-specific survival (HR 1.28; 95% CI 1.03–1.66) [19]. Similarly, in pT3 disease, nephrectomy delays longer than 10 weeks from diagnosis have been associated with worse overall survival (adjusted HR 1.13; 95% CI 1.04–1.24) [17]. These findings suggest that prolonged preoperative waiting times – as observed in our study- may be associated with adverse oncologic outcomes, particularly in advanced-stage disease.

Few studies have examined the pandemic’s effects on hospital length of hospital stay in KC patients. A recent Indian study reported a 15-day longer mean hospitalization during COVID-19, attributed to clearance protocols such as preoperative testing, chest CTs, and increased complication rates [1], while others linked longer stays to more advanced disease [23, 31]. In contrast, we found no significant difference in median LOH between the study periods. This could reflect offsetting factors: several studies document shortened hospital stays (up to 26%) during the pandemic due to strategies minimizing infection risk and preserving bed capacity, including expedited discharges and telemonitoring [32, 33]. It is plausible that hospital policy adaptations may have counterbalanced potential COVID-related delays in our setting.

Patient and tumor characteristics have been increasingly recognized as influential factors in the waiting times for diagnosis and treatment of patients with KC, particularly during the COVID-19 pandemic when healthcare resources were redirected [3, 5, 6, 23]. Previous studies have demonstrated that patients with advanced (T3 or T4) tumors experience markedly shorter waiting times compared to those with lower-stage disease [27, 34]. A recent systematic review reported that tumor aggressiveness and severity consistently drive accelerated care, whereas localized or asymptomatic cancers often experience longer diagnostic and waiting intervals [5].

In line with this, we found that advanced-stage disease was a protective factor against prolonged TDI, however only in the pre-pandemic period but not during the pandemic. This appears to be corroborated by international reports suggesting that between 20% and 31.4% of individuals avoided healthcare during the pandemic due to infection fear, even for potentially urgent symptoms [1, 5, 24, 35]. The phenomenon indicates the need for patient education, to recognize kidney cancer symptoms and seek timely care irrespective of external healthcare disruptions.

Advanced-stage disease was also a protective predictor against prolonged THI, but only during the pandemic. This aligns with findings that healthcare systems triage and prioritize care based on clinical urgency and likely reflects prioritization policies adapted to pandemic-related resource limitations [6, 23, 31]. In our cohort, this translated into the preferential fast-tracking of patients with advanced disease—likely associated with more overt symptoms or aggressive features—for urgent surgical intervention, thereby bypassing bottlenecks affecting earlier-stage cases. In contrast, asymptomatic patients with localized masses were more likely to experience “non-urgent” deferrals [8]. This pattern of care is in line with the European Association of Urology guidance, which put emphasis on the prioritization of symptomatic and high-stage renal tumors to avoid clinically meaningful delays [36].

Since THI was not influenced by the stage of cancer before the pandemic, it implied that once patients entered care, tumor stage had limited influence on treatment delay, indicating comparable urgency in managing both early and advanced cases thereafter. Another possible factor is that more complex surgery for advanced cancer may require additional preparation, potentially offsetting any time advantage [31], which appears to be supported by our findings that advanced cancer was not a protective predictor of prolonged TTI in either study periods. Since no studies have yet compared KC waiting times before and during the pandemic by cancer stage, further research is required to identify underlying reasons and to contextualize our findings.

### Limitations

This study has limitations. It was conducted at a single tertiary center, potentially limiting generalizability. Generalizability is further constrained in the analysis of TDI, as this was restricted to the symptomatic patient subgroup, representing only a small proportion of the total cohort. Direct comparison with international waiting times should be approached cautiously, as patient pathways, healthcare organization, and pandemic policies differ between countries. Pandemic-related changes in health-seeking behavior (delays) and case mode distribution (fewer incidentally detected, more symptomatic cases) possibly influenced our findings. Additionally, determination of symptom onset relied on patient self-report, which is subjective and may introduce recall bias. Furthermore, although advanced tumor stage was identified as a significant predictor of shorter waiting times, potential interactions with other demographic or clinical variables were not assessed and warrant further investigation in future studies. Finally, this study lacks long-term survival and oncological outcome data, which prevents a direct assessment of how the reported waiting times impacted actual patient prognosis.

## Conclusion

To our knowledge, this study is among the first to assess the impact of COVID-19 on kidney cancer waiting times in Central and Eastern Europe. Our findings showed that while TTI and THI remained stable, TDI increased significantly among symptomatic patients during the pandemic, indicating that delays occurred primarily before the point of healthcare entry rather than during subsequent clinical management. These results highlight the importance of patient education and engagement to discourage delayed healthcare-seeking, even during periods of system disruption.

Advanced-stage disease was identified as a protective predictor against prolonged TDI before the pandemic and against prolonged THI during the pandemic. This pattern suggests that patients delayed seeking care irrespective of symptom severity during COVID-19, whereas advanced cases were appropriately prioritized once within the healthcare system.

As a secondary observation, median healthcare-related waiting times in our setting were longer than those typically reported internationally, suggesting potential regional challenges in healthcare access. This underscores systemic differences in care pathways and the need for targeted interventions to reduce delays. Further research is warranted to clarify the underlying causes of these disparities.

Overall, this study provides regionally relevant insights into kidney cancer care delivery, with implications for health-system optimization and patient-centered strategies during and beyond public health crises.

## Data Availability

The original contributions presented in the study are included in the article/Supplementary Material, further inquiries can be directed to the corresponding author.

## References

[1] Nerli RB Gautam S Ghagane SC Rai S . Impact of COVID-19 on treatment in patients with renal cell carcinoma. Arch Razi Inst (2024) 79(5):1091–7. 10.32592/ari.2024.79.5.1091 40292050 PMC12018751

[2] Gupta A Patil A Patel D Singh AG Ganpule AP Sabnis RB Stage migration in renal malignancies in COVID era: a single-center analysis. Indian J Surg Oncol (2023) 14(4):1–6. 10.1007/s13193-023-01771-3 37363712 PMC10187501

[3] Ou W Wang C Un H Guo S Xiao H Huang B Impact of time-to-surgery on the prognosis of patients with T1 renal cell carcinoma: implications for the COVID-19 pandemic. J Clin Med (2022) 11(24):7517. 10.3390/jcm11247517 36556133 PMC9786871

[4] Edge R Meyers J Tiernan G Li Z Schiavuzzi A Chan P Cancer care disruption and reorganisation during the COVID-19 pandemic in Australia: a patient, carer and healthcare worker perspective. PLoS One (2021) 16(9):e0257420. 10.1371/journal.pone.0257420 34534231 PMC8448370

[5] Chan VW Tan WS Leow JJ Ong WLK Chiu PKF Pratik G Delayed surgery for localised and metastatic renal cell carcinoma: a systematic review and meta-analysis for the COVID-19 pandemic. World J Urol (2021) 39(12):4295–303. 10.1007/s00345-021-03734-1 34031748 PMC8143063

[6] Janes WCI Fagan MG Andrews JM Harvey DR Warden GM Johnston PH Impact of the COVID-19 pandemic on diagnosis of renal cell carcinoma and disease stage at presentation. Can Urol Assoc J (2024) 18(4):E113–e119. 10.5489/cuaj.8519 38381938 PMC11034969

[7] Bekele BB Alhaffar BA Wasnik RN Sándor J . The effect of the COVID-19 pandemic on the social inequalities of health care use in Hungary: a nationally representative cross-sectional study. Int J Environ Res Public Health (2022) 19(4):2258. 10.3390/ijerph19042258 35206447 PMC8872504

[8] Rumi L Szántó Á Bányai D Szabó É Zemplényi A Bellyei S Changes in the characteristics of kidney cancer detection during the COVID-19 pandemic. Cancers (Basel) (2025) 17(13):2150. 10.3390/cancers17132150 40647450 PMC12248619

[9] Capitanio U Bensalah K Bex A Boorjian SA Bray F Coleman J Epidemiology of renal cell carcinoma. Eur Urol (2019) 75(1):74–84. 10.1016/j.eururo.2018.08.036 30243799 PMC8397918

[10] Hollingsworth JM Miller DC Daignault S Hollenbeck BK . Rising incidence of small renal masses: a need to reassess treatment effect. J Natl Cancer Inst (2006) 98(18):1331–4. 10.1093/jnci/djj362 16985252

[11] Peired AJ Campi R Angelotti ML Antonelli G Conte C Lazzeri E Sex and gender differences in kidney cancer: clinical and experimental evidence. Cancers (Basel) (2021) 13(18):4588. 10.3390/cancers13184588 34572815 PMC8466874

[12] Hu SL Weiss RH . Management of the incidental kidney mass in the nephrology clinic. Clin J Am Soc Nephrol (2018) 13(9):1407–9. 10.2215/cjn.00860118 29653957 PMC6140565

[13] Bahadoram S Davoodi M Hassanzadeh S Bahadoram M Barahman M Mafakher L . Renal cell carcinoma: an overview of the epidemiology, diagnosis, and treatment. G Ital Nefrol (2022) 39(3):2022-vol3. 35819037

[14] Znaor A Lortet-Tieulent J Laversanne M Jemal A Bray F . International variations and trends in renal cell carcinoma incidence and mortality. Eur Urol (2015) 67(3):519–30. 10.1016/j.eururo.2014.10.002 25449206

[15] Salih FM Omar SS Hamza HT Namiq KS Ameen HRM Rasul KI Long-term outcomes and survival rates of renal cell carcinoma patients in erbil, Iraq: a follow-up study. BMC Cancer (2025) 25(1):384. 10.1186/s12885-024-13040-9 40033222 PMC11874839

[16] Stec AA Coons BJ Chang SS Cookson MS Herrell SD Smith JA Waiting time from initial urological consultation to nephrectomy for renal cell carcinoma--does it affect survival? J Urol (2008) 179(6):2152–7. 10.1016/j.juro.2008.01.111 18423724 PMC2953870

[17] Zeng J Batai K Lee BR . Nephrectomy delay of more than 10 weeks from diagnosis is associated with decreased overall survival in pT3 RCC. J Kidney Cancer VHL (2021) 8(2):27–33. 10.15586/jkcvhl.v8i2.125 34178583 PMC8211570

[18] Macleod U Mitchell ED Burgess C Macdonald S Ramirez AJ . Risk factors for delayed presentation and referral of symptomatic cancer: evidence for common cancers. Br J Cancer (2009) 101(Suppl. 2):S92–s101. 10.1038/sj.bjc.6605398 19956172 PMC2790698

[19] Jia K Wang M Wang Y Yuan D Huang L Li K Oncologic outcomes of surgical delay by interval and tumor size in T1b-T2aN0M0 renal cell carcinoma. World J Surg Oncol (2025) 23(1):409. 10.1186/s12957-025-04067-8 41174724 PMC12577136

[20] Zhang X Elsaid MI DeGraffinreid C Champion VL Paskett ED Impact of COVID-19 on Behaviors across the Cancer Control Continuum in Ohio group. Impact of the COVID-19 pandemic on cancer screening delays. J Clin Oncol (2023) 41(17):3194–202. 10.1200/jco.22.01704 36735899 PMC10256430

[21] Keim-Malpass J Vavolizza RD Cohn WF Kennedy EM Showalter SL . Cancer screening and treatment delays during the COVID-19 pandemic and the role of health literacy in care Re-engagement: findings from an NCI-designated comprehensive cancer center sample. J Cancer Educ (2023) 38(5):1405–12. 10.1007/s13187-023-02312-w 37202597 PMC10195653

[22] Geng S Zhang L Zhang Q Wu Y . Ethical dilemmas for palliative care nurses: systematic review. BMJ Support Palliat Care (2024). 10.1136/spcare-2023-004742 38538036

[23] Gürel A Baylan B Özen A Keleş I Öztekin U Demirbaş A Comparison of renal cell cancer surgery during the COVID-19 pandemic with prepandemic period, Turkey multicenter study. Bull Urooncology. (2022) 21(4):119–23. 10.4274/uob.galenos.2021.2021.11.3

[24] Wang Z Tang Y Cui Y Guan H Cui X Liu Y Delay in seeking health care from community residents during a time with low prevalence of COVID-19: a cross-sectional national survey in China. Front Public Health (2023) 11:1100715. 10.3389/fpubh.2023.1100715 36895687 PMC9989024

[25] Keskin A Yorulmaz EM Donmez K Ozcan S Kose O Gorgel SN Impact of the coronavirus disease 2019 (COVID-19) pandemic on tumor stage progression in urological malignancies: a comparative study. Front Urol (2025) 5:1619185. 10.3389/fruro.2025.1619185 40843453 PMC12364627

[26] Yildirim H Bins AD van den Hurk C van Moorselaar RJA van Oijen MGH Bex A The impact of the COVID-19 pandemic on renal cancer care. World J Urol (2024) 42(1):231. 10.1007/s00345-024-04925-2 38613582 PMC11016011

[27] Martínez CH Martin P Chalasani V Williams AK Luke PPW Izawa JI How long can patients with renal cell carcinoma wait for surgery without compromising pathological outcomes? Can Urol Assoc J (2011) 5(6):E148–51. 10.5489/cuaj.10035 21388587 PMC3235220

[28] Zhao F Liu X Zhang C Zhu H Qi N . Mortality increases when radical nephrectomy is delayed more than 60 days for T3 renal cell carcinoma. Technol Cancer Res Treat (2021) 20:15330338211043963. 10.1177/15330338211043963 34595976 PMC8489746

[29] Comission E . Country cancer profile (hungary) (2025). Available online at: https://www.oecd.org/content/dam/oecd/en/publications/reports/2025/02/eu-country-cancer-profile-hungary-2025_d482f082/344b5f49-en.pdf (Accessed January 12, 2025).

[30] Papp ZM Szakács L Hajivandi SS Kalina I Varga E Kiss G Impact of a targeted project for shortening of imaging diagnostic waiting time in patients with suspected oncological diseases in Hungary. Medicina (Kaunas) (2023) 59(1):153. 10.3390/medicina59010153 36676777 PMC9865166

[31] Collins PM Madden A O'Connell C Omer SA Shakeel Inder M Casey RG Urological service provision during the COVID-19 period: the experience from an Irish tertiary centre. Ir J Med Sci (2021) 190(2):455–60. 10.1007/s11845-020-02352-x 32856269 PMC7451224

[32] Wang KY McNeely EL Dhanjani SA Raad M Puvanesarajah V Neuman BJ COVID-19 significantly impacted hospital length of stay and discharge patterns for adult spinal deformity patients. Spine (Phila Pa 1976) (2021) 46(22):1551–6. 10.1097/brs.0000000000004204 34431833 PMC8552912

[33] Kilaru AS Porges SB Grossman L Delgado MK Morgan AU Chaiyachati KH An accelerated hospital observation pathway to reduce length of stay for patients with COVID-19. Am J Manag Care (2022) 28(6):262–8. 10.37765/ajmc.2022.88789 35738222

[34] Qi N Zhao F Liu X Wei W Wang J . Safety of prolonged wait time for nephrectomy for clinically localized renal cell carcinoma. Front Oncol (2021) 11:617383. 10.3389/fonc.2021.617383 33859936 PMC8042291

[35] Splinter MJ Velek P Ikram MK Kieboom BCT Peeters RP Bindels PJE Prevalence and determinants of healthcare avoidance during the COVID-19 pandemic: a population-based cross-sectional study. Plos Med (2021) 18(11):e1003854. 10.1371/journal.pmed.1003854 34813591 PMC8610236

[36] Bex A Ghanem YA Albiges L Bonn S Campi R Capitanio U European association of urology guidelines on renal cell carcinoma: the 2025 update. Eur Urol (2025) 87(6):683–96. 10.1016/j.eururo.2025.02.020 40118739

