# Supplementary material for: Delayed diagnostic imaging but stable treatment initiation for kidney cancer during the COVID-19 pandemic: a Hungarian cohort study
Source: Pathol Oncol Res. 2026 May 13;32:1612411. doi: 10.3389/pore.2026.1612411 (PMC13212254; doi:10.3389/pore.2026.1612411)
Supplement: Supplementary file 1 [file Table1.docx]

*Supplementary Table 1: Demographic and clinical factors: A) Sex B) Age, C) Residence type D) Distance from Clinical Center E) CCI score and D) Stage demonstrating no statistically significant relationship with waiting times (TDI, THI-S, TTI-S, THI, TTI) and LOH in the pre-pandemic and pandemic periods.*

**A)**

| Time to initial diagnostic imaging  TDI | | | Gender | |
| --- | --- | --- | --- | --- |
|  |  |  | **Male** | **Female** |
| Before COVID-19  [p= 0.389] | **n [%]** | **≤ median** | 10 [7.2%] | 7 [10.8%] |
|  |  | **> median** | 129 [92.8%] | 58 [89.2%] |
| During COVID-19  [p= 0.132] | **n [%]** | **≤ median** | 12 [10.7%] | 4 [4.8%] |
|  |  | **> median** | 100 [89.3%] | 80 [95.2%] |

| Time to histopathological  diagnosis THI | | | Gender | |
| --- | --- | --- | --- | --- |
|  |  |  | **Male** | **Female** |
| Before COVID-19  [p=0.240] | **n [%]** | **≤ median** | 70 [50.4%] | 27 [41.5%] |
|  |  | **> median** | 69 [49.6%] | 38 [58.5%] |
| During COVID-19  [p=0.591] | **n [%]** | **≤ median** | 57 [50.9%] | 46 [54.8%] |
|  |  | **> median** | 55 [49.1%] | 38 [45.2%] |

| Time to treatment  TTI | | | Gender | |
| --- | --- | --- | --- | --- |
|  |  |  | **Male** | **Female** |
| Before COVID-19  [p=0.167] | **n [%]** | **≤ median** | 70 [50.4%] | 26 [40.0%] |
|  |  | **> median** | 69 [49.6%] | 39 [60.0%] |
| During COVID-19  [p=0.649] | **n [%]** | **≤ median** | 61 [54.5%] | 43 [51.2%] |
|  |  | **> median** | 51 [45.5%] | 41 [48.8%] |

| Length of hospitalization  LOH | | | Gender | |
| --- | --- | --- | --- | --- |
|  |  |  | **Male** | **Female** |
| Before COVID-19  [p=0.283] | **n [%]** | **≤ median** | 90 [64.7%] | 37 [56.9%] |
|  |  | **> median** | 49 [35.3%] | 28 [43.1%] |
| During COVID-19  [p=0.262] | **n [%]** | **≤ median** | 59 [52.7%] | 51 [60.7%] |
|  |  | **> median** | 53 [47.3%] | 33 [39.3%] |

**B)**

| TDI  Time to initial diagnostic imaging | | | Effect of age | | | |
| --- | --- | --- | --- | --- | --- | --- |
|  |  |  | **0 – 49** | **50 – 59** | **51 – 69** | **≥ 70** |
| Before COVID-19  [p=0.669] | **n [%]** | **≤ median** | 2 [6.7%] | 6 [11.1%] | 6 [9.7%] | 3 [5.2%] |
|  |  | **> median** | 28 [93.3%] | 48 [88.9%] | 56 [90.3%] | 55 [94.8%] |
| During COVID-19  [p=0.695] | **n [%]** | **≤ median** | 3 [11.1%] | 6 [10.5%] | 5 [7.0%] | 2 [4.9%] |
|  |  | **> median** | 24 [88.9%] | 51 [89.5%] | 66 [93.0%] | 39 [95.1%] |

| THI Time to histopathological  diagnosis | | | Effect of age | | | |
| --- | --- | --- | --- | --- | --- | --- |
|  |  |  | **0 – 49** | **50 – 59** | **51 – 69** | **≥ 70** |
| Before COVID-19  [p= 0.179] | **n [%]** | **≤ median** | 19 [63.3%] | 21 [38.9%] | 28 [45.2%] | 29 [50.0%] |
|  |  | **> median** | 11 [36.7%] | 33 [61.1%] | 34 [54.8%] | 29 [50.0%] |
| During COVID-19  [p= 0.461] | **n [%]** | **≤ median** | 16 [59.3%] | 32 [56.1%] | 32 [45.1%] | 23 [56.1%] |
|  |  | **> median** | 11 [40.7%] | 25 [43.9%] | 39 [54.9%] | 18 [43.9%] |

| TTI  Time to treatment | | | Effect of age | | | |
| --- | --- | --- | --- | --- | --- | --- |
|  |  |  | **0 – 49** | **50 – 59** | **51 – 69** | **≥ 70** |
| Before COVID-19  [p= 0.093] | **n [%]** | **≤ median** | 20 [66.7%] | 21 [38.9%] | 27 [43.5%] | 28 [48.3%] |
|  |  | **> median** | 10 [33.3%] | 33 [61.1%] | 35 [56.5%] | 30 [51.7%] |
| During COVID-19  [p= 0.424] | **n [%]** | **≤ median** | 17 [63.0%] | 30 [52.6%] | 33 [46.5%] | 24 [58.5%] |
|  |  | **> median** | 10 [37.0%] | 27 [47.4%] | 38 [53.5%] | 17 [41.5%] |

| LOH  Length of hospitalization | | | Effect of age | | | |
| --- | --- | --- | --- | --- | --- | --- |
|  |  |  | **0 – 49** | **50 – 59** | **51 – 69** | **≥ 70** |
| Before COVID-19  [p= 0.861] | **n [%]** | **≤ median** | 19 [63.3%] | 34 [63.0%] | 36 [58.1%] | 38 [65.5%] |
|  |  | **> median** | 11 [36.7%] | 20 [37.0%] | 26 [41.9%] | 20 [34.5%] |
| During COVID-19  [p= 0.386] | **n [%]** | **≤ median** | 17 [63.0%] | 29 [50.9%] | 44 [62.0%] | 20 [48.8%] |
|  |  | **> median** | 10 [37.0%] | 28 [49.1%] | 27 [38.0%] | 21 [51.2%] |

**C)**

| Time to initial diagnostic imaging  TDI | | | Residence type | | |
| --- | --- | --- | --- | --- | --- |
|  |  |  | **County seat** | **City** | **Other** |
| Before COVID-19  [p= 0.646] | **n [%]** | **≤ median** | 6 [11.1%] | 5 [8.3%] | 6 [6.7%] |
|  |  | **> median** | 48 [88.9%] | 55 [91.7%] | 84 [93.3%] |
| During COVID-19  [p= 0.213] | **n [%]** | **≤ median** | 5 [9.3%] | 3 [4.0%] | 8 [11.9%] |
|  |  | **> median** | 49 [90.7%] | 72 [96.0%] | 59 [88.1%] |

| Time to histopathological  diagnosis THI | | | Residence type | | |
| --- | --- | --- | --- | --- | --- |
|  |  |  | **County seat** | **City** | **Other** |
| Before COVID-19  [p= 0.297] | **n [%]** | **≤ median** | 30 [55.6%] | 29 [48.3%] | 38 [42.2%] |
|  |  | **> median** | 24 [44.4%] | 31 [51.7%] | 52 [57.8%] |
| During COVID-19  [p= 0.342] | **n [%]** | **≤ median** | 24 [44.4%] | 43 [57.3%] | 36 [53.7%] |
|  |  | **> median** | 30 [55.6%] | 32 [42.7%] | 31 [46.3%] |

| Time to treatment  TTI | | | Residence type | | |
| --- | --- | --- | --- | --- | --- |
|  |  |  | **County seat** | **City** | **Other** |
| Before COVID-19  [p= 0.045] | **n [%]** | **≤ median** | 33 [61.1%] | 27 [45.0%] | 36 [40.0%] |
|  |  | **> median** | 21 [38.9%] | 33 [55.0%] | 54 [60.0%] |
| During COVID-19  [p= 0.328] | **n [%]** | **≤ median** | 24 [44.4%] | 42 [56.0%] | 38 [56.7%] |
|  |  | **> median** | 30 [55.6%] | 33 [44.0%] | 29 [43.3%] |

| Length of hospitalization  LOH | | | Residence type | | |
| --- | --- | --- | --- | --- | --- |
|  |  |  | **County seat** | **City** | **Other** |
| Before COVID-19  [p= 0.901] | **n [%]** | **≤ median** | 35 [64.8%] | 37 [61.7%] | 55 [61.1%] |
|  |  | **> median** | 19 [35.2%] | 23 [38.3%] | 35 [38.9%] |
| During COVID-19  [p= 0.884] | **n [%]** | **≤ median** | 29 [53.7%] | 42 [56.0%] | 39 [58.2%] |
|  |  | **> median** | 25 [46.3%] | 33 [44.0%] | 28 [41.8%] |

**D)**

| Time to initial diagnostic imaging  TDI | | | Distance from clinical center | |
| --- | --- | --- | --- | --- |
|  |  |  | **≤ 40 km** | **> 40 km** |
| Before COVID-19  [p= 0.040] | **n [%]** | **≤ median** | 11 [13.1%] | 6 [5.0%] |
|  |  | **> median** | 73 [86.9%] | 114 [95.0%] |
| During COVID-19  [p= 0.099] | **n [%]** | **≤ median** | 5 [5.0%] | 11 [11.5%] |
|  |  | **> median** | 95 [95.0%] | 85 [88.5%] |

| Time to histopathological  diagnosis THI | | | Distance from clinical center | |
| --- | --- | --- | --- | --- |
|  |  |  | **≤ 40 km** | **> 40 km** |
| Before COVID-19  [p= 0.987] | **n [%]** | **≤ median** | 40 [47.6%] | 57 [47.5%] |
|  |  | **> median** | 44 [52.4%] | 63 [52.5%] |
| During COVID-19  [p= 0.898] | **n [%]** | **≤ median** | 53 [53.0%] | 50 [52.1%] |
|  |  | **> median** | 47 [47.0%] | 46 [47.9%] |

| Time to treatment  TTI | | | Distance from clinical center | |
| --- | --- | --- | --- | --- |
|  |  |  | **≤ 40 km** | **> 40 km** |
| Before COVID-19  [p= 0.880] | **n [%]** | **≤ median** | 39 [46.4%] | 57 [47.5%] |
|  |  | **> median** | 45 [53.6%] | 63 [52.5%] |
| During COVID-19  [p= 0.788] | **n [%]** | **≤ median** | 54 [54.0%] | 50 [52.1%] |
|  |  | **> median** | 46 [46.0%] | 46 [47.9%] |

| Length of hospitalization  LOH | | | Distance from clinical center | |
| --- | --- | --- | --- | --- |
|  |  |  | **≤ 40 km** | **> 40 km** |
| Before COVID-19  [p= 0.427] | **n [%]** | **≤ median** | 55 [65.5%] | 72 [60.0%] |
|  |  | **> median** | 29 [34.5%] | 48 [40.0%] |
| During COVID-19  [p= 0.235] | **n [%]** | **≤ median** | 52 [52.0%] | 58 [60.4%] |
|  |  | **> median** | 48 [48.0%] | 38 [39.6%] |

**E)**

| Time to initial diagnostic imaging  TDI | | | CCI – Charlson Comorbidity Index | |
| --- | --- | --- | --- | --- |
|  |  |  | **≤ 4** | **≥ 5** |
| Before COVID-19  [p= 0.669] | **n [%]** | **≤ median** | 8 [9.3%] | 9 [7.6%] |
|  |  | **> median** | 78 [90.7%] | 109 [92.4%] |
| During COVID-19  [p= 0.382] | **n [%]** | **≤ median** | 10 [9.8%] | 6 [6.4%] |
|  |  | **> median** | 92 [90.2%] | 88 [93.6%] |

| Time to histopathological  diagnosis THI | | | CCI – Charlson Comorbidity Index | |
| --- | --- | --- | --- | --- |
|  |  |  | **≤ 4** | **≥ 5** |
| Before COVID-19  [p= 0.800] | **n [%]** | **≤ median** | 40 [46.5%] | 57 [48.3%] |
|  |  | **> median** | 46 [53.5%] | 61 [51.7%] |
| During COVID-19  [p= 0.331] | **n [%]** | **≤ median** | 57 [55.9%] | 46 [48.9%] |
|  |  | **> median** | 45 [44.1%] | 48 [51.1%] |

| Time to treatment  TTI | | | CCI – Charlson Comorbidity Index | |
| --- | --- | --- | --- | --- |
|  |  |  | **≤ 4** | **≥ 5** |
| Before COVID-19  [p= 0.880] | **n [%]** | **≤ median** | 41 [47.7%] | 55 [46.6%] |
|  |  | **> median** | 45 [52.3%] | 63 [53.4%] |
| During COVID-19  [p= 0.162] | **n [%]** | **≤ median** | 59 [57.8%] | 45 [47.9%] |
|  |  | **> median** | 43 [42.2%] | 49 [52.1%] |

| Length of hospitalization  LOH | | | CCI – Charlson Comorbidity Index | |
| --- | --- | --- | --- | --- |
|  |  |  | **≤ 4** | **≥ 5** |
| Before COVID-19  [p= 0.893] | **n [%]** | **≤ median** | 54 [62.8%] | 73 [61.9%] |
|  |  | **> median** | 32 [37.2%] | 45 [38.1%] |
| During COVID-19  [p= 0.720] | **n [%]** | **≤ median** | 56 [54.9%] | 54 [57.4%] |
|  |  | **> median** | 46 [45.1%] | 40 [42.6%] |

**F)**

| Time to treatment  TTI | | | Stage | |
| --- | --- | --- | --- | --- |
|  |  |  | **Early** | **Advanced** |
| Before COVID-19  [p= 0.143] | **n [%]** | **≤ median** | 71 [44.4%] | 25 [56.8%] |
|  |  | **> median** | 89 [55.6%] | 19 [43.2%] |
| During COVID-19  [p= 0.186] | **n [%]** | **≤ median** | 74 [50.3%] | 30 [61.2%] |
|  |  | **> median** | 73 [49.7%] | 19 [38.8%] |

| Length of hospitalization  LOH | | | Stage | |
| --- | --- | --- | --- | --- |
|  |  |  | **Early** | **Advanced** |
| Before COVID-19  [p= 0.123] | **n [%]** | **≤ median** | 104 [65.0%] | 23 [52.3%] |
|  |  | **> median** | 56 [35.0%] | 21 [47.7%] |
| During COVID-19  [p= 0.868] | **n [%]** | **≤ median** | 83 [56.5%] | 27 [55.1%] |
|  |  | **> median** | 64 [43.5%] | 22 [44.9%] |
